# TRPV6 and Calbindin-D9k-expression and localization in the bovine uterus and placenta during pregnancy

**DOI:** 10.1186/1477-7827-10-66

**Published:** 2012-08-29

**Authors:** Nele Sprekeler, Mariusz P Kowalewski, Alois Boos

**Affiliations:** 1 Institute of Veterinary Anatomy, Vetsuisse Faculty, University of Zurich, Winterthurerstrasse 260, Zurich, 8057, Switzerland

**Keywords:** TRPV6, Calbindin-D9k, Cow, Placenta, Uterus, Pregnancy

## Abstract

**Background:**

Transient receptor potential channel type 6 (TRPV6) and Calbindin-D9k (CaBP-9k) are involved in the active calcium (Ca2+) transport mechanism in many tissues including placenta and uterus, suggesting a role in the establishment and maintenance of pregnancy. Moreover, TRPV6 and CaBP-9k seem to support the materno-fetal Ca2+ transport that is crucial for fetal Ca2+ homeostasis, bone growth and development. However, it is unknown if these proteins are also involved in the aetiology of pathologies associated with parturition in cows, such as retained fetal membranes (RFM). The aim of the current study was to create an expression profile of uterine and placentomal TRPV6 and CaBP-9k mRNAs and proteins during pregnancy and postpartum in cows with and without fetal membrane release.

**Methods:**

Uteri and placentomes of 27 cows in different stages of pregnancy and placentomes of cows with and without RFM were collected. Protein and mRNA expression of TRPV6 and CaBP-9k was investigated by real-time PCR, immunohistochemistry and Western blot.

**Results:**

In the uterine endometrium, highest TRPV6 and CaBP-9k expression was found in the last trimester of pregnancy, with a particular increase of protein in the glandular epithelium. In the placentomes, a gradual increase in TRPV6 mRNA was detectable towards parturition, while protein expression did not change significantly. Placentomal CaBP-9k expression did not change significantly throughout pregnancy but immunohistochemistry revealed an increase in staining intensity in the maternal crypt epithelium. Immunohistochemical, stronger placental CaBP-9k signals were seen in animals with RFM compared to animals with an undisturbed fetal membrane release, while protein levels, measured by Western blot analyses did not change significantly.

**Conclusions:**

The results of the present study demonstrate a dynamic expression of TRPV6 and CaBP-9k during pregnancy in the bovine uterine endometrium and placentomes, suggesting a functional role for these proteins in Ca2+ metabolism during pregnancy. The temporal and spatial expression patterns indicate that TRPV6 and CaBP-9k may be involved in materno-fetal Ca2+ transport, mainly through an interplacentomal transport, and that both proteins may participate in physiological processes that are crucial for fetal and placental development. However, neither TRPV6 nor CaBP-9k seem to be causative in the retention of fetal membranes.

## Background

The presence of Ca^2+^ in female reproductive tissues is essential during pregnancy. Uterine Ca^2+^ ions are thought to be involved in the establishment and maintenance of pregnancy, possibly by controlling myometrial activity and by influencing the secretion of extra-cellular matrix components [1-3]. Furthermore, materno-fetal transport of Ca^2+^ is crucial for fetal development, e.g. bone mineralization, neuromuscular activities, and blood coagulation [4,5]. In the placenta, Ca^2+^ functions as an important second messenger that regulates several cellular processes such as transcription, proliferation, differentiation, necrosis and apoptosis [6]. During pregnancy, the fetal requirement for Ca^2+^ increases strikingly, especially in the last trimester of gestation: the Ca^2+^ content of bovine fetuses increased from 3 g/kg at d 100 postmating to 8 g/kg at d 280, indicating a high level of materno-fetal Ca^2+^ transfer [7]. This Ca^2+^ must be transported against a concentration gradient from the mother to the fetus [8], thus an active transport mechanism is required.

TRPV6 and CaBP-9k (also known as S100 calcium binding protein G) are crucial elements in the active transport mechanism of mammals. TRPV6 is an epithelial Ca^2+^ channel, localized in the apical membrane of epithelial cells in several tissues, including small intestine, kidney, uterus and placenta [9], which mediates Ca^2+^ uptake into the cell. CaBP-9k is known to facilitate intracellular Ca^2+^ transport from the apical to the basolateral cell membrane and functions additionally as an intracellular buffer of Ca^2+^ in many tissues, such as intestine, bone, uterus and placenta [10]. The expression, and to a certain extent, the regulation of CaBP-9k and TRPV6 in female reproductive tissues is well documented in several mammalian species, such as rat, mouse, pig and human [5,11-14]. Unlike in Ca^2+^-absorbing organs like the duodenum, where the expression of these proteins is regulated mainly via vitamin-D, their expression in the female reproductive system seems to be regulated via the steroid hormones progesterone and estrogens [2,11,12]. Interestingly, there are distinct variations in the expression and regulation of both proteins among different mammals, which explains their species-specific, dynamic expression patterns during the estrous cycle and pregnancy [1,2,11].

The clinical importance of TRPV6 in female reproductive tissues has been shown using TRPV6-knockout mice [4]. These animals showed reduced fertility, the materno-fetal Ca^2+^ transport was 40% lower and fetal Ca^2+^ accumulation was reduced compared to wild type mice. The physiological role of uterine CaBP-9k appears to be the regulation of myometrial activity, while in the placenta CaBP-9k is thought to be involved in the regulation of the materno-fetal Ca^2+^ transport [3,12].

Taken together, several findings indicate that TRPV6 and CaBP-9k may play an important role in the establishment and maintenance of pregnancy and the materno-fetal Ca^2+^ transport in mammals. Thus an undisturbed Ca^2+^ metabolism in the reproductive tissues is crucial for fertility and fetal health.

So far, however, the expression of uterine and placentomal TRPV6 expression has not been studied during pregnancy in the cow. Moreover, it is unknown if TRPV6 or CaBP-9k are involved in the development of pathologies related to pregnancy and parturition. The expression of bovine CaBP-9k was investigated in the bovine uterus during the estrous cycle [15], where it was mainly detected during the luteal phase, and placentomal CaBP-9k was examined in the second half of pregnancy, where its expression level increased during the last trimester, correlating with the increased fetal Ca^2+^ demand [16].

This study was performed to obtain further information about the uterine and placentomal Ca^2+^ metabolism at different stages of pregnancy in the cow. Therefore, the temporal and spatial expression patterns of TRPV6 and CaBP-9k protein and mRNA in the uterine endometrium and in the placentomes were examined.

Retained fetal membranes (RFM) in cows are described as an anomaly in the normal release of the fetal membranes within 12 hours after calving [17]. The mechanism leading to RFM is still unclear. Factors such as nutritional deficiencies, inflammation of the placenta after parturition, disturbances in maturation of the fetomaternal unit and in apoptotic processes have all been implicated in the aetiology of RFM [18].

Since Ca^2+^ plays such an important role in many cellular processes concerning placental maturation, such as proliferation, differentiation, transcription or apoptosis [6], it is also a matter of interest if TRPV6 and CaBP-9k may be involved in the detachment of the placenta postpartum. Therefore, this study further investigated the placental expression of TRPV6 and CaBP-9k postpartum in animals that discharged their fetal membranes within 12 hours after parturition and animals that did not.

## Methods

### Animals

Uteri of 45 pregnant Holstein-Friesian cows were collected at the local abattoir and three or four at each month 2–9 of gestation were selected at random for the present study. Within 30 minutes after the animals were killed, they were eviscerated, the pregnant uteri were opened and the crown-rump lengths of the 45 fetuses were recorded to estimate fetal age as described by Boos et al. [19].

The study also examined placental specimens of five spontaneously calving postpartum cows that discharged the fetal membranes (DFM) within 12 hours after expulsion of the fetuses and of five animals that retained fetal membranes (RFM). These cattle were members of the university dairy herd or patients from the Clinic for Bovine Obstetrics and Gynaecology, University of Veterinary Medicine Hannover, Foundation.

Animal experiments were approved by the administration of the district Hannover, and performed according to the German Law for the Protection of Animals (TierschG) and the recommendations of the German Society of Laboratory Animal Science (GV-SOLAS).

### Tissue sampling and processing

At the abattoir, at least two randomly selected and comparatively large placentomes and two segments of the interplacentomal uterine wall including adherent allantochorion were excised from each animal. Macroscopically visible caruncles, the adhering allantochorion and the surrounding segment of the uterine wall were excised from the uterine horn ipsilateral to the corpus luteum in early pregnant cows. Only a single place tome each -but no uterine wall- was collected from ten cows within one hour after the expulsion of their calf. For excision of placentomes from live cows, an elongated effeminator according to Reisinger (1906), modified by Richter (1936) (Hauptner, Solingen) was used.

A centrally located cross section of approximately 10 mm in thickness was performed on selected placentomes. The sections were cut in pieces of 10x10mm and three samples each were selected for further processing. These were: (1) uterine wall oriented – concave side – tissue cube, i.e., placental tissue including maternal plate and placental stalk material; (2) allantoic sac oriented – convex side – tissue cube, i.e., placental tissue including fetal plate material, and finally (3) tissue cube collected from the periphery – equatorial – also including fetal plate and placental tissue. The specimens were oriented and mounted in Tissue-Tek II® (Miles, Elkhart, Ind., USA) on cork lamellae, snap frozen and stored at −80°C.

For immunohistochemistry, tissue samples were rinsed in 0.9% saline, fixed in 4% neutral buffered formalin for 24 hours and finally embedded in paraffin (Histowax, Leica) as previously described [19].

### RNA isolation and quantitative real-time (Taqman) PCR

Total RNA from bovine uterine wall and placentomes from the 2^nd^ to 9^th^ month of pregnancy (n = 3 animals /month) and placentomes of postpartum cows with (RFM, n = 5) and without retained fetal membranes (DFM, n = 6) was isolated using TRIzol Reagent (Invitrogen, Carlsbad, CA) according to the manufacturer’s protocol. RNA concentration was measured by spectrophotometry (SmartSpec^TM^ Plus Spectrophotometer, Biorad, Reinach, Switzerland). Purity and quality of mRNA were determined by optical density (OD) measurement. The OD 260/280 ratio of all samples was greater than 1.8. Then, 200 ng of total RNA was DNase treated (DNase I recombinant, RNase free, Roche Diagnostics, Indianapolis, USA) and reverse transcribed using the Gold RNA PCR Core Kit (Gene Amp, Applied Biosystems, Foster City, CA). The RT reaction was run for 8 minutes at 21°C, 15 minutes at 45°C and 5 minutes at 99°C.

Real Time (Taqman) PCR was performed using the ABI 7500 Fast Real-Time PCR System (Applied Biosystems, Rotkreuz, Switzerland), as described previously [20]. In brief, samples were amplified in duplicates in a 25-μl reaction mixture containing 12.5 μl FastStart Universal Probe Master (Roche Diagnostics, Mannheim, Germany), 300 nM of each primer, 200 nM Taqman probe and 5 μl cDNA. Autoclaved water instead of RNA and the so-called RT minus control were used as negative controls. Primers and 6-carboxyfluorescein (6-FAM)- and 6-carboxytetramethyl-rhodamine (TAMRA)-labelled probes were designed using Primer Express Software (Applied Biosystems) and ordered from Eurogentec, B-4102 Seraing, Belgium. For primer and probe sequences see Table 1. The expression levels of TRPV6 and CaBP-9k were quantified by the comparative CT method, calculated relative to the expression of the reference gene GAPDH and normalized to the calibrator. The sample with the lowest level of the respective target gene transcripts was used as the calibrator. The efficiency of the PCR reactions was verified using the CT slope method according to the instructions of the manufacturers of the ABI 7500 Fast Real-Time PCR System. The specificity of the selected PCR products was confirmed by sequencing (Microsynths, Switzerland). 

**Table 1:** Sequences of primers and probes

	**Primer sequence**	**Accession #**
TRPV6 forward	5 -CAAGGAGCCCATGACATCTGA-3	
TRPV6 reverse	5 -CAGGGCTTTCACGAGGTTCA-3	NM_001206189.1
TRPV6 probe	5 -CGGCCCTGCACATAGCAGTCATGAA-3	
CaBP-9k forward	5 -TCTCCAGAAGAACTGAAGGGC-3	
CaBP-9k reverse	5 -CCAACACCTGGAATTCTTCG-3	NM_174257.2
CaBP-9k probe	5 -TCCAAGCACCCTCGATGAGCTTTTTGA-3	
GAPDH forward	5 -GCGATACTCACTCTTCTACCTTCGA-3	
GAPDH reverse	5 -TCGTACCAGGAAATGAGC TTGAC-3	NM_001034034.1
GAPDH probe	5 -CTGGCATTGCCCTCAACGACCACTT-3	

### Immunohistochemical analysis

Paraffin-embedded tissue samples were cut at 3 μm. Sections were dew axed using xylol and rehydrated through serial dilutions of ethanol to water and rinsed in water. Antigen retrieval was performed by boiling the sections in 10 mM citrate buffer pH 6.0 in a microwave oven (600 W, 3 x 5 minutes) and rinsing in Tris-buffered saline (TBS, buffer stock solution: 6.1 g Trizma base, 50 ml H_2_0 and 37 ml 1 N HCl, diluted with H_2_O to 1000 ml solution, pH adjusted to 7.6; working solution: 100 ml buffer stock solution plus 900 ml 0.85% saline). Endogenous peroxidase activity was inhibited by incubating the sections in 0.3% H_2_O_2_ in methanol for 15 minutes. Non-specific binding was blocked with 10% normal goat serum. The tissue sections were incubated at 4°C overnight with the primary antibody. The expression of TRPV6 was detected with a rabbit polyclonal anti-TRPV6 antibody (ACC-036, Alomone Labs, Israel) at a 1:200 dilution, which reacts with the intracellular C-terminus. The expression of CaBP-9k was detected with a rabbit polyclonal anti-CaBP-9k-antibody (Swant, Bellinzona, Switzerland) at a dilution of 1:5000, which was produced against rat recombinant Calbindin-D9k protein. As negative controls, tissue sections were incubated with an isotype specific rabbit IgG (Vector Laboratories, Burlingame, CA, USA) and with TBS instead of the primary antibody.

After rinsing in TBS for 3x10 minutes, the biotinylated secondary antibody (PowerVision Poly-HRP IHC Detection System, goat anti-Rabbit IgG) was added in dilution of 1:200 and sections were incubated for 30 minutes at 23°C. After washing in TBS for 2x10 minutes, the tissue sections were incubated for 30 minutes with StreptABComplex/HRP Duett (Dako, Glostrup, DK), and Chromogen diaminobenzidine tetrahydrochloride (Liquid DAB + Substrate, Dako, Baar, Switzerland) was added. Before being automatically coverslipped (RCM 2000®; Medite) in Pertex® (Medite), the sections were counterstained with haematoxylin.

### Western blot

The tissue samples (n = 3 animals/month) were homogenised in NET-2 buffer (50 mM Tris–HCl, pH 7.4, 300 mM NaCl, 0.05% Nonidet P-40 [21] containing 1 μl/ml protease inhibitor (Sigma-Aldrich, Buchs, Switzerland) and centrifuged (1000 *g*) for 10 minutes at 4°C. The clarified supernatants were removed, and the protein concentrations were determined by the Bradford method (Bradford, 1976) (Bio-Rad Laboratories, Reinach, Switzerland). Samples were diluted with NET-2 buffer and sample buffer, containing β-mercaptoethanol, and incubated for 5 minutes at 95°C.

Proteins were separated on SDS-Gel (TRPV6: 10% polyacrylamide, CaBP-9k: 12% polyacrylamide) and transferred onto PVDF membranes. For the deglycosylation of TRPV6, the proteins were denatured at 100°C and incubated with N-glycosidase F (PNGase F, New England Biolabs, Frankfurt, Germany) at 37°C for 1 hour, before separation by SDS-Gel, according to the manufacturer’s protocol. After blocking the membranes in 5% fat-free milk in PBS-T (Tris-buffered saline with 0.025% Tween-20) for one hour, the membranes were incubated overnight at 4°C with the primary antibodies diluted in 2.5% milk/PBS-T. The antibodies were identical to those used for immunohistochemistry, diluted 1:500 (TRPV6) and 1:5000 (CaBP-9k). As a negative control for the TRPV6 antibody, samples were preincubated with a specific control antigen (Alomone labs, Israel).

Afterwards, the blots were washed in PBS-T three times for 10 minutes and incubated with the secondary peroxidase-conjugated antibody for one hour at 23°C (anti-rabbit IgG (H + L) HRP conjugate, Promega, Dübendorf, Switzerland). Membranes were washed five times in PBS-T for 10 minutes. Protein detection was performed by chemiluminescence (Immun-Star HRP Substrate Kit, Bio-Rad) and exposed to LAS-3000 (Fujifilm). The membranes were reblotted with anti-GAPDH antibody at a 1:5000 dilution as the loading control. The optical density of the TRPV6, CaBP-9k and GAPDH bands was quantified using ImageJ software. The values are presented as the ratio of TRPV6 and CaBP-9k optical density to the corresponding GAPDH optical density.

### Statistics

The data sets of real-time and Western blot were tested for normality using the Kolmogorov-Smirnov Test [22]. Small deviations from normality were observed in the dataset of CaBP-9k mRNA expression in the placenta. Therefore, these data were analyzed by a nonparametric one-way analysis of variance using the Kruskal-Wallis test, followed by Dunn`s post test. The remaining datasets were analyzed using a parametric one way analyses of variance (ANOVA) followed by Dunnetts comparison test, where the second month was taken as the control group. Real-time and Western blot data sets of animals with retained fetal membranes in comparison to animals that discharged the fetal membranes were analyzed by an unpaired one-tailed t-test. For all of the statistical analyses, the software programme GraphPad Prism 5.0 (GraphPad Software Inc., San Diego, CA, USA) was used. P < 0.05 was considered statistically significant. Data are presented as means ± SD.

## Results

### Expression of TRPV6 and Calbindin-D9k mRNA in the uterus and placenta during pregnancy

**Figure 1:**
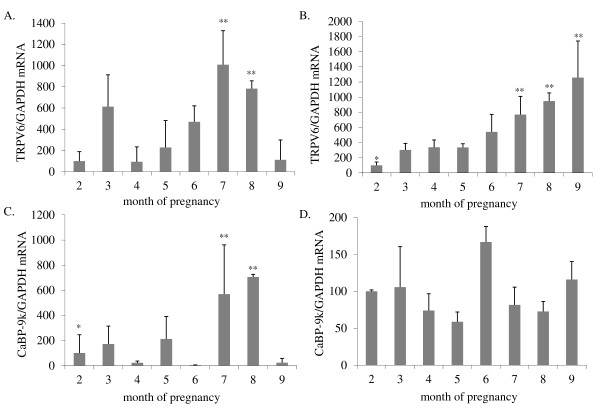
**Uterine and placentomal TRPV6 and CaBP-9k mRNA expression.** TRPV6 and CaBP-9k mRNA expression in the intercaruncular wall (Figure 1A, C) and in the placenta (n ≥ 3 animals/month) (Figure 1B, D) from the 2^nd^ to 9^th^ months of pregnancy, determined by real-time PCR. Bars with ** differ significantly (p < 0.05) compared to the second month (*). Data were analysed using a parametric one-way analysis of variance (ANOVA), followed by Dunnett’s comparison test and are presented as means ± SD. The nonparametric dataset of placental CaBP-9k was analysed using the Kruskal-Wallis test, followed by Dunn`s post test.

TRPV6 and CaBP-9k mRNA expression in the uterine wall and in the placentomes from the 2^nd^ to 9^th^ months of pregnancy was investigated by quantitative real-time (Taqman) PCR. In the uterine wall, TRPV6 mRNA was detected throughout pregnancy with the highest levels in the 7^th^ and 8^th^ months (p < 0.05) (Figure 1A). Placental TRPV6 expression levels increased from the 2^nd^ month to the highest levels in the 7^th^, 8^th^ and 9^th^ months of pregnancy (p < 0.05) (Figure 1B).

Interplacentomal uterine CaBP-9k mRNA expression was detected throughout pregnancy with a significant increase in the 7^th^ and 8^th^ months (p < 0.05) (Figure 1C).

In the placentomes CaBP-9k mRNA was detected throughout the pregnancy without any significant changes (p > 0.05) (Figure 1D).

### Localisation of TRPV6 protein in the uterus and placenta during pregnancy

**Figure 2:**
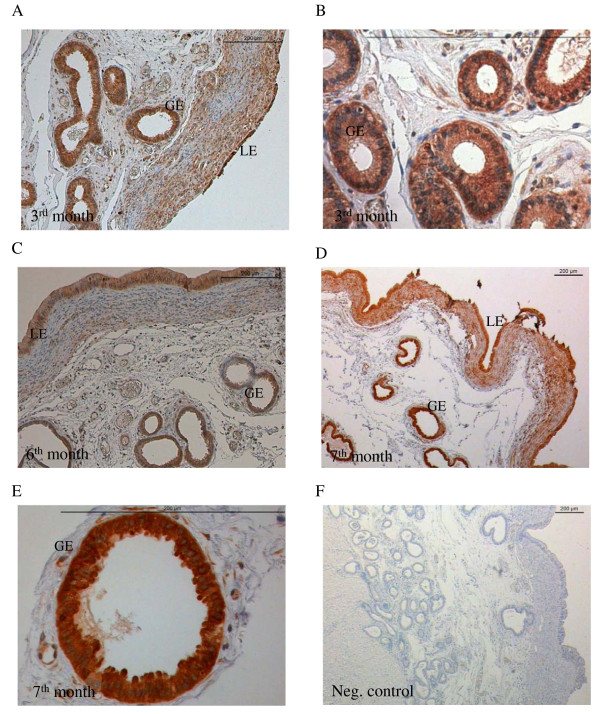
**Immunolabelling of TRPV6 in the uterine endometrium.** Localisation of uterine TRPV6 during pregnancy with strong labelling in the glandular (GE) and luminal epithelia (LE). Figure 2F shows a negative control, without anti-TRPV6 treatment. Scale bars indicate 200 μm.

**Figure 3:**
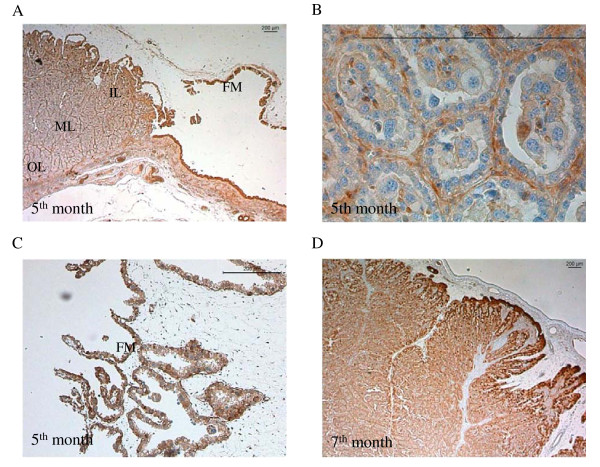
**Immunolabelling of TRPV6 in the placentomes and adherent fetal membranes.** Localisation of TRPV6 during pregnancy in the placenta and adherent membranes with strong staining in the inner layer (IL) of the place tome **(A, D)** and the adherent fetal membranes (FM) **(A, C).** Considerably weaker staining in the middle (ML) and outer labyrinth (OL) layers **(A, B).** Scale bars indicate 200 μm.

Immunohistochemistry was performed to determine the localization of TRPV6 in the uterine intercaruncular wall and in the placentomes during pregnancy (Figures 2, 3). In the uterus TRPV6 protein was detected in the luminal and in the glandular epithelium and revealed the strongest labelling in the glandular epithelium in the 3^rd^ month and from the 7^th^ month until parturition (Figure 2).

In the placenta, TRPV6 was primarily expressed in the inner layer of the placentomes throughout pregnancy. Considerably lower staining was found in the middle and outer layers of the labyrinth (Figures 3A, D). Strongest labelling was seen in the adherent fetal membranes (Figure 3A, C). The distribution at the cellular level was diffuse (Figure 3B).

### Localisation of CaBP-9k protein in the uterus and placenta during pregnancy

**Figure 4:**
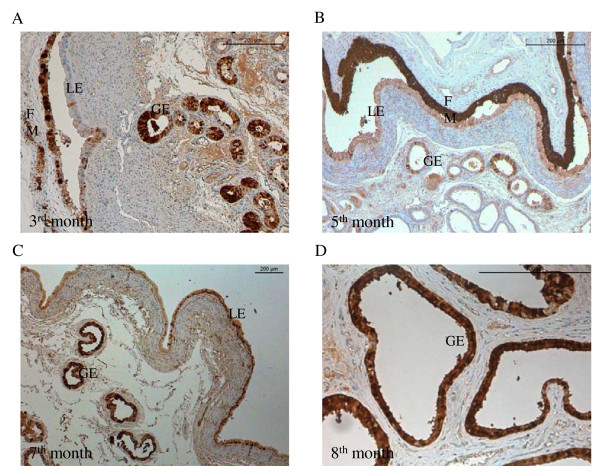
**Immunolabelling of CaBP-9k protein in the uterine endometrium.** Localisation of CaBP-9k in the glandular (GE) and luminal epithelia (LE) during pregnancy** (A-D). **Strongest staining was detected in the fetal membranes (FM) **(A, B).** Scale bars indicate 200 μm.

**Figure 5:**
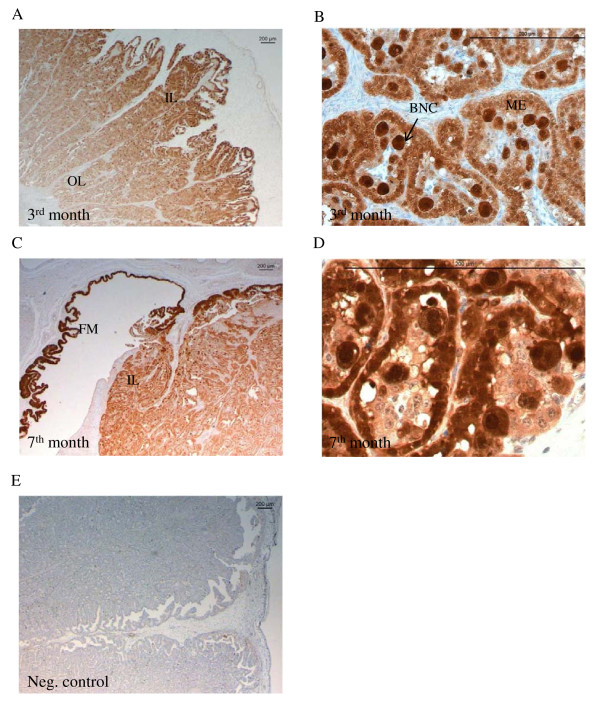
**Immunolabelling of CaBP-9k in placentomes and adherent fetal membranes.** Localisation of CaBP-9k during pregnancy with strong signals in the inner layer (IL) of the place tome and in the fetal membranes (FM) compared to the outer labyrinth layer (OL) **(A, C). **Strong staining in the binucleate trophoblast cells (BNC) and in the maternal epithelium (ME) inside the place tome **(B, C). **Figure 2F shows a negative control, without anti-TRPV6 treatment. Scale bars indicate 200 μm.

The localization of CaBP-9k in the uterine endometrium and in the placentomes was investigated by immunohistochemistry (Figures 4, 5). In the endometrium, CaBP-9k was detected in the luminal and glandular epithelium. The strongest staining intensity was visible in the glandular epithelium of the 7^th^ and 8^th^ months of pregnancy (Figure 4C, D). Furthermore the trophoblast epithelium of the adherent fetal membranes was strongly stained (Figure 4A, B).

Placental CaBP-9k protein was mainly detected in the luminal, inner layer and in the fetal membranes (Figure 5A, C). Staining intensity decreased towards the outer labyrinth layer. On the cellular level, CaBP-9k was expressed in the cytoplasm and nuclei of the maternal crypt epithelium and in the uni- and binucleate fetal cells. The strongest staining was found in the binucleate cells and in the maternal crypt epithelium. In the latter the staining intensity increased towards parturition (Figure 5B, D).

### Western blot analyses of TRPV6 and CaBP-9k protein in the uterus and placenta during pregnancy

**Figure 6:**
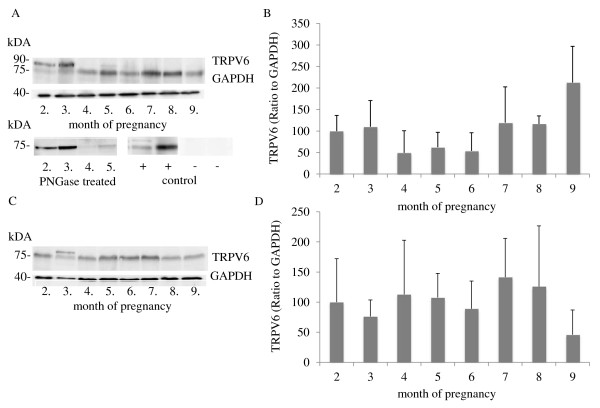
**Western blot analyses of endometrial and placentomal TRPV6 protein during pregnancy. A)** Representative Western blot of TRPV6 protein expression in the uterine wall (n = 3 animals/month). Bands of approximately 75 kDa were detected. An additional band of approximately 90 kDa was seen in the 2^nd^ and 3^rd^ months. After incubation with N-glycosidase F (PNGase F), only the smaller band of 75 kDa was seen, demonstrating that the other band is the glycosylated variant of TRPV6. As a negative control, samples were preincubated with a control antigen. **B)** Relative expression of TRPV6 to GAPDH was determined by densitometry using ImageJ software. No significant changes were detected (p > 0.05). **C)** Representative Western blot analyses of TRPV6 in the placenta from 2^nd^ to 9^th^ months of pregnancy (n = 3 animals/month). **D)** The expression of TRPV6 relative to GAPDH was determined by measuring the optical density using ImageJ software. No significant changes (p > 0.05) were detected. Data were analysed using a parametric one-way analysis of variance (ANOVA), followed by Dunnett’s comparison test, where the second month was taken as the control group, and are presented as means ± SD.

**Figure 7:**
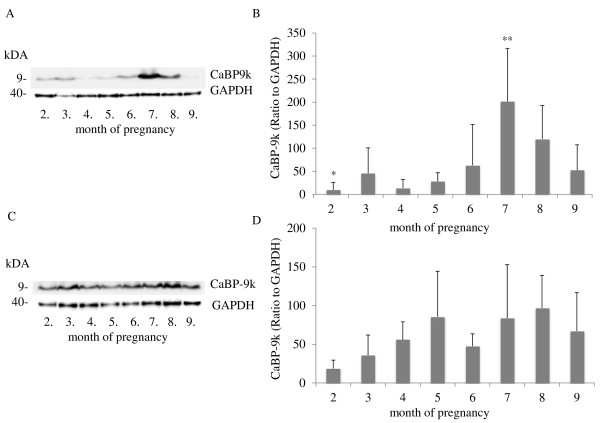
**Western blot analyses of endometrial and placental CaBP-9k protein during pregnancy. A)** Representative Western blot of CaBP-9k in the uterine wall (n = 3 animals/month). Bands of approximately 9 kDa were detected. **B)** The highest expression relative to GAPDH was found in the 7^th^ month of pregnancy, determined by measuring the optical density using ImageJ software. Bars with ** differ significantly (p < 0.05) compared to the 2^nd^ month (*). **C)** Representative Western blot of CaBP-9k in the placenta (n = 3 animals/month). **D)** Relative expression levels of CaBP-9k to GAPDH were determined by densitometry using ImageJ software. Significant changes in the expression levels were not detected (p > 0.05). Data were analysed using a parametric one-way analysis of variance (ANOVA), followed by Dunnett’s comparison test, where the second month was taken as the control group, and are presented as means ± SD.

In addition to the protein localization, the expression of TRPV6 and CaBP-9k protein in the uterine wall and in the placentomes was further investigated by Western blot analyses (Figures 6, 7). In both the uterine and placental tissues, bands of TRPV6 with a molecular mass of 75 kDa were detected from the 2^nd^ to the 9^th^ months without significant changes in expression levels (Figure 6). In some animals a second band of approximately 90 kDa was detected in the 2^nd^ and/or 3^rd^ months, which represents the glycosylated variation of TRPV6.

Bands of CaBP-9k protein with a molecular weight of 9 kDa were detected in the uterine and placental tissues (Figure 7). The highest (p < 0.05) expression levels were found in the uterine endometrium in the 7^th^ month of pregnancy (Figure 7B). In the placenta, no significant changes were detectable during pregnancy (Figure 7D).

### Expression and localization of TRPV6 and CaBP-9k in the placenta post partum in cows with and without retention of fetal membranes

**Figure 8:**
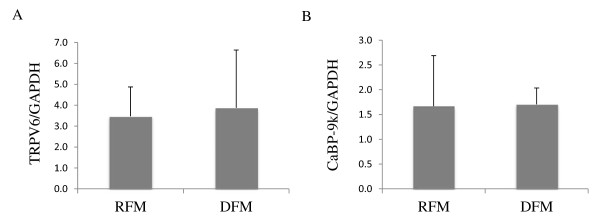
**Placental TRPV6 and CaBP-9k mRNA expression post partum in cows with and without retained fetal membranes.** Detection of TRPV6 (A) and Calbindin-D9k (CaBP-9k) (B) mRNA levels in the placenta of postpartum cows that retained the fetal membranes for more than 12 hours (RFM; n = 5) and cows that discharged the fetal membranes (DFM; n = 6), measured by real-time PCR (Taqman). No significant changes were observed (p > 0.05). Data were analyzed by an unpaired one-tailed t-test and are presented as means ± SD.

The placental mRNA levels of TRPV6 and CaBP-9k of animals that retained fetal membranes postpartum compared to animals that discharged fetal membranes were examined by real-time PCR. Neither the TRPV6 nor the CaBP-9k expression levels showed significant differences between the groups (p > 0.5) (Figure 8).

**Figure 9:**
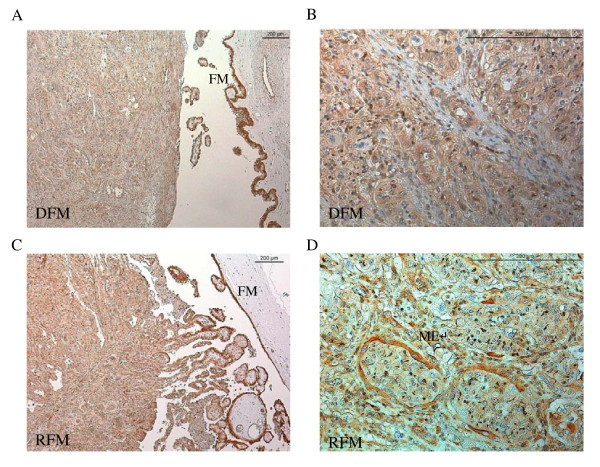
**Localisation of TRPV6 protein in the placentomes and fetal membranes in cows with (RFM) (C, D) and without retained fetal membranes (DFM) (A, B).** Strongest labeling of TRPV6 protein was seen in the fetal membranes (FM) of all animals and in the maternal crypt epithelium (ME) of cows with RFM** (D).**

**Figure 10:**
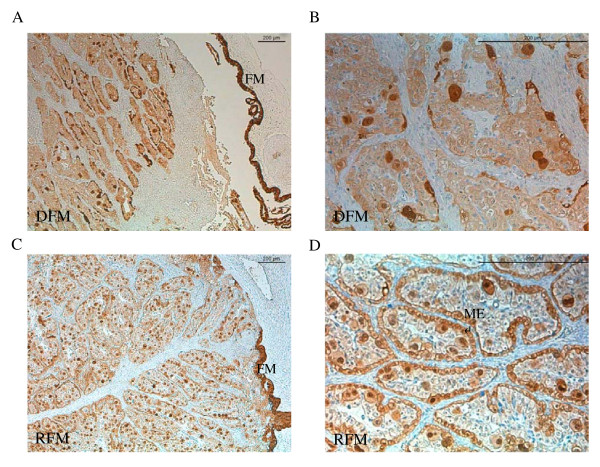
**Localisation of placental CaBP-9k protein post partum in cows with (RFM) (C, D) and without retained fetal membranes (DFM) (A, B).** Strong staining was detected in the fetal membranes (FM) of all investigated animals and in the maternal crypt epithelium (ME) of animals with retained fetal membranes **(D) **and in the binucleate trophoblast cells (BNC) of all animals.

Immunolabelling of TRPV6 and CaBP-9k protein was performed to localise these proteins in the placentomes of animals that retained and animals that discharged the fetal mambranes (Figures 9, 10). The strongest labelling of both proteins was detected in the fetal membranes in both groups. In the place tome, TRPV6 was found in the maternal part of the place to me and in the trophoblast cells of both groups (Figure 9B, D). Strong staining of TRPV6 was found in the maternal crypt epithelium of animals that retained their fetal membranes (Figure 9D). Similar observations were made in the distribution of CaBP-9k in the place to me. Besides the strong staining in the fetal membranes (Figure 10A, C), strongest signals were localized in the binucleate trophoblastic cells and in the maternal crypt epithelium of cows that retained fetal membranes (Figure 10D). The uninucleate cells were also stained, but the intensity was considerably weaker.

**Figure 11:**
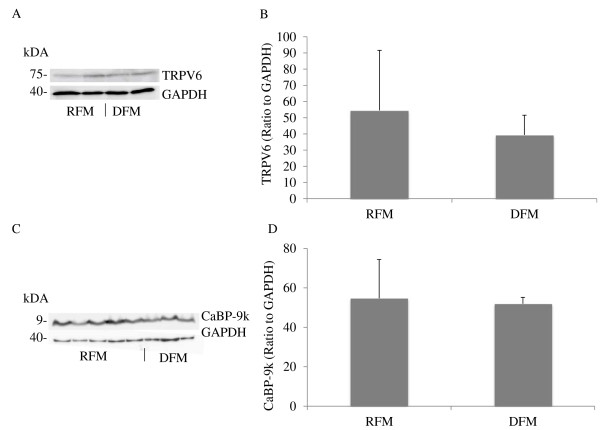
**Western blot analyses of placental TRPV6 and CaBP-9k postpartum in cows with (RFM) and without retained fetal membranes (DFM). A)** Representative Western blot of TRPV6 in the placenta. Bands of approximately 75 kDa were detected in all animals. **B)** Relative expression of TRPV6 was determined by measuring the optical density relative to GAPDH using ImageJ software. Significant changes in expression levels were not detected (p > 0.05). **C)** Representative Western blot of CaBP-9k. **D)** The relative expression levels to GAPDH were determined by densitometry using ImageJ software. No significant changes were detected (p > 0.05). Data were analyzed by an unpaired one-tailed t-test and are presented as means ± SD.

In the Western blot analyses, no significant differences in TRPV6 and CaBP-9k expression were detected between the groups (p > 0.5) (Figure 11).

## Discussion

The results of the current study show for the first time that TRPV6 is expressed in time- and cell type specific patterns during different stages of pregnancy in the uterine endometrium and placentomes in the cow, indicating a functional role for this protein in Ca^2+^ metabolism during pregnancy. Furthermore, the expression patterns of CaBP-9k as an important intracellular Ca^2+^ transporter and buffer were investigated to check for possible colocalization and functional correlation between both factors.

In other species, such as rodents and pigs, TRPV6 seems to participate in the implantation process, in the maintenance of pregnancy, in the fetal Ca^2+^ supply and in fetal development [1,2,4,11]. CaBP-9k may play a major role in the implantation process, in the regulation of myometrial activity and in the regulation of materno-fetal Ca^2+^ transport [12,23,24]. The expression of CaBP-9k in the bovine endometrium and placentomes has been described previously [16,25], however, it has not been shown to correlate with TRPV6 expression nor with pathological conditions such as fetal membrane retention.

In the current study, uterine expression of both TRPV6 and CaBP-9k mRNA was highest in the 7^th^ and 8^th^ months of pregnancy, the time of enhanced fetal growth and skeletal mineralization [7]. The localization of both proteins in the glandular and luminal uterine epithelium indicates an increase in Ca^2+^ transport through or demand within these epithelia, which is met by high expression of TRPV6 and CaBP-9k.

Ca^2+^ plays a key physiological role in many cellular processes, including in secretory and exocytose processes [26], so the increased demand in the glandular and luminal epithelia may correlate with the secretory activity of the epithelial cells. The glandular and luminal epithelia are responsible, among other functions, for the production and secretion of histiotrophe, which is essential for fetal development [27] and growth. Thus, the localization and the expression of TRPV6 and CaBP-9k in the uterine endometrium, mainly in the last trimester which is the time of enhanced fetal growth, indicate a functional role in secretory activities and therefore in fetal nourishment and development.

This idea is further supported by the high expression of TRPV6 and CaBP-9k in the fetal membranes, indicating a high interplacentomally active Ca^2+^ transport into the fetus, which previously has been demonstrated in-vitro by Using-chamber studies in sheep [28]. The colocalization of both proteins suggests a functional correlation between TRPV6 and CaBP-9k in the bovine endometrium, which is well documented for other epithelia such as the small intestine [29].

In the present study, the results of the Western blot analyses did not show any significant time-dependent changes in the expression of either of the proteins with the exception of uterine CaBP-9k. Moreover, distinct individual variations were detected between animals at the same stages of pregnancy, as evidenced by the high standard variations (SD) of the data obtained. The immunohistochemistry results illustrate that the measured protein (and mRNA) levels are very dependent on the anatomical composition of the samples taken for the Western blot analyses and that even small changes in anatomical structure and composition can be responsible for these variations. This could also be a reason for the divergence between the measured mRNA and protein levels, particularly since the results of the immunohistochemistry demonstrate increased protein expression in the 7^th^ and 8^th^ months. Nevertheless, the Western blot data show a clear trend of expression patterns and provide additional useful information. Furthermore, the antibody used for the TRPV6 Western blot analyses detected two proteins of approximately 75 and 90kDA. Since TRPV6 is a glycoprotein [30], analyses of glycosylation were performed. After incubation in the presence of N-glycosidase F, only the smaller variation of 75 kDa was detectable, demonstrating that the 90 kDa band represents the glycosylated variation of TRPV6. Inside the place tome, TRPV6 was mainly expressed in the luminal, inner layer of the labyrinth. While a gradual increase in placental TRPV6 mRNA was found towards parturition, the protein expression levels did not change significantly throughout pregnancy. A possible role for the discrepancy between mRNA and protein levels may be post-transcriptional regulation, or the above-mentioned possibility of small divergences in anatomical structure. A similar placental increase of TRPV6 mRNA was described in rats [2], while mouse placental TRPV6 expression peaks in the middle but not at the end of pregnancy [1]. The expression patterns of TRPV6 in the bovine placenta indicate an influence on placental Ca^2+^ metabolism, but their precise function remains unclear.

The considerably higher expression of TRPV6 protein in the interplacentomal fetal membranes compared to its expression in the placentomes provides evidence that materno-fetal Ca^2+^ transport occurs mainly in the interplacentomal regions and TRPV6 seems to play a crucial role in this process in the cow. Similar results are documented in mice, where the intra- and extra-placental yolk sac is described to be the site of materno-fetal Ca^2+^ transport in which TRPV6 seems to be a crucial element [4].

The high expression of CaBP-9k and the colocalization with TRPV6 in the fetal membranes supports this theory. It has been demonstrated in earlier studies that CaBP-9k levels in the ruminant interplacental trophoblasts are eight to ten times higher than in the placentomal region [25], which confirms our results and supports the idea of a facilitated diffusion of Ca^2+^ through the fetal membranes to supply the enhanced fetal need for Ca^2+^, especially in the last trimester of pregnancy.

The intraplacetomal distribution of CaBP-9k, mainly at the luminal side of the labyrinth, correlates with TRPV6 localization, but on the cellular level there are distinct variations. CaBP-9k is mostly expressed in the binucleate trophoblast cells (BNC), as described previously [16] and in the maternal crypt epithelium, while the distribution of TRPV6 is more diffuse. Interestingly, the staining intensity of CaBP-9k in the maternal epithelium increases towards parturition. The distribution of CaBP-9k and its changes indicate a functional role in placentomal Ca^2+^ metabolism. As described before, Ca^2+^ is important for many cell physiological processes, which are also crucial for placental development and degradation [6]. Thus, the high expression of CaBP-9k in the binucleate trophoblast cells may reflect the intracellular need for Ca^2+^ to support the active metabolism of these cells. In the ruminant placenta, the BNCs are, among other cells, responsible for the synthesis of hormones and proteins, for migration and cell-cell-interactions [31], all processes which are Ca^2+^ dependent [32,33].

Furthermore, the high CaBP-9k expression in the maternal crypt epithelium possibly reflects the increased Ca^2+^ requirement for cell-specific processes, such as integrin-mediated placentation, placental growth and placental maturation [19]. The important role of intracellular Ca^2+^ as a mediator of processes concerning placental maturation in the last trimester, such as degradation, apoptosis and separation, may explain the increase of CaBP-9k expression by the maternal epithelium in the last trimester of pregnancy. Based on this theory, it is possible that disturbed Ca^2+^ cell metabolism influences maturation of the placenta and apoptotic degradation of the maternal crypt epithelium, which could lead to a disturbance in release of the fetal membranes after parturition [19,34]. Therefore, this study further examined the expression patterns of TRPV6 and CaBP-9k in animals that retained the fetal membranes compared to animals that discharged the fetal membranes within 12 hours after parturition. In the placentomes, both proteins were mainly detected in the maternal epithelium, CaBP-9k additionally in the trophoblast cells, especially in the binucleate cells. This localization explains the higher expression in animals that retained the fetal membranes, since the maternal epithelium is largely intact in these animals while it is strongly reduced in cows with normal fetal membrane release. Thus, the idea that a deficiency of TRPV6 or CaBP-9k expression leads to a disturbance in release of fetal membranes could not be confirmed. Expression of both proteins reflects the status of the epithelia involvement and is rather a consequence than the cause of the retention of fetal membranes.

## Conclusions

The results of the present study demonstrate for the first time that TRPV6 is dynamically expressed in the bovine uterine endometrium and in the placentomes during pregnancy, indicating a functional role in Ca^2+^ metabolism during gestation.

The expression of TRPV6 and CaBP-9k in the uterine glandular epithelium, in the placental maternal epithelium and the fetal chorionic membranes suggests that these proteins are involved in materno-fetal Ca^2+^ transfer mainly through interplacental transport, especially in the last trimester of pregnancy, a time of enhanced fetal growth. Their distinct expression in the uterine glandular epithelium indicates that TRPV6 and CaBP-9k participate in uterine secretory processes, which are crucial for fetal nourishment and development.

Due to their increased expression in the maternal epithelium and in the binucleate trophoblastic cells in the placentomes, TRPV6 and CaBP-9k may function as important elements in controlling placental development, maturation and degradation through the regulation of intracellular Ca^2+^ metabolism. However, neither TRPV6 nor CaBP-9k seem to be causative in the retention of fetal membranes.

## Competing interests

The authors declare that they have no competing interests.

## Authors’ contributions

NS was involved in the design of the study, in the coordination and performance of the experiments, in the evaluation and interpretation of the data and the drafting of the manuscript. MPK was involved in the experimental part of the project and revision of the manuscript. AB was involved in the design of the study, in the sampling procedure and supervision of the project, and was involved in drafting the manuscript. All authors read and approved the final manuscript.
